# “Research participants want to feel they are better off than they were before research was introduced to them”: engaging cameroonian rural plantation populations in HIV research

**DOI:** 10.1186/1472-698X-12-8

**Published:** 2012-06-22

**Authors:** Emmanuel Kiawi, Eleanor McLellan-Lemal, Jembia Mosoko, Kata Chillag, Pratima L Raghunathan

**Affiliations:** 1 Centers for Disease Control and Prevention-Cameroon, c/o US Embassy, BP 817, Yaoundé, Cameroon; 2 Division of HIV/AIDS Prevention, Centers for Disease Control and Prevention, Atlanta, USA; 3 Division of Global HIV/AIDS, Center for Global Health, Centers for Disease Control and Prevention, Mutengene, Cameroon; 4 Division of Global HIV/AIDS, Center for Global Health, Centers for Disease Control and Prevention, Kigali, Rwanda

## Abstract

**Background:**

During a period of evolving international consensus on how to engage communities in research, facilitators and barriers to participation in HIV prevention research were explored in a rural plantation community in the coastal region of Cameroon.

**Methods:**

A formative rapid assessment using structured observations, focus group discussions (FGD), and key informant interviews (KIIs) was conducted with a purposive non-probabilistic sample of plantation workers and their household members. Eligibility criteria included living or working >1 year within the plantation community and age >18 years. Both rapid and in-depth techniques were used to complete thematic analysis.

**Results:**

Sixty-five persons participated in the study (6 FGDs and 12 KIIs). Participants viewed malaria and gastrointestinal conditions as more common health concerns than HIV. They identified three factors as contributing to HIV risk: concurrent sexual relationships, sex work, and infrequent condom use. Interviewees perceived that the community would participate in HIV research if it is designed to: (1) improve community welfare, (2) provide comprehensive health services and treatment for illnesses, (3) protect the personal information of participants, especially those who test positive for HIV, (4) provide participant incentives, (5) incorporate community input, and (6) minimize disruptions to “everyday life”. Barriers to participation included: (1) fear of HIV testing, (2) mistrust of researchers given possible disrespect or intolerance of plantation community life and lack of concern for communication, (3) time commitment demands, (3) medical care and treatment that would be difficult or costly to access, and (4) life disruptions along with potential requirements for changes in behaviour (i.e., engage in or abstain from alcohol use and sex activities).

**Conclusions:**

Consistent with UNAIDS guidelines for good participatory practice in HIV prevention research, study participants placed a high premium on researchers’ politeness, trust, respect, communication, tolerance and empathy towards their community. Plantation community members viewed provision of comprehensive health services as an important community benefit likely to enhance HIV research participation.

## Background

HIV represents the heaviest burden of disease contributing to current morbidity and mortality rates among adults in Cameroon [1]. With a population of 19.5 million, it is estimated that 141 new HIV infections occur each day in the country [2]. The most recent Cameroon Demographic and Health Survey, for which data is currently available, showed that in 2004 HIV prevalence was estimated at 5.4% among adults [1,3], with significant variations by region, age, sex and place of residence [3]. For this same time period, the Northwest (8.7%), East (8.6%) and Southwest (8.0%) regions showed the highest rates with urban residents 21–39 years of age accounting for persons at greatest risk of becoming infected [2]. Similar to the rest of sub-Saharan Africa, prevalence for men is lower than for women (4.1% compared to 6.8%) [3]^.^ However, rates are relatively higher among employed men (5.1% compared to 2.1% for unemployed men) [2], suggesting the workplace may be an appropriate focus for HIV prevention activities among men.

To date, most HIV prevention research has focused on high-risk urban populations, namely sex workers, truck drivers, persons residing along the Cameroon-Chad oil pipeline, students, and uniformed men and women [4,5]. Fewer studies have emphasized rural populations despite their vulnerabilities [6]. Recent findings suggest that rural populations in sub-Saharan Africa have begun to account for an increased percentage of new cases of HIV infection [1]. Because they are likely to be poor, less educated, uninformed, isolated, lack decision-making authority, and to experience a multitude of barriers in accessing basic health services, rural populations are more susceptible to ill health and disease, including HIV infection [7].

A large number of agricultural plantations (palm oil, rubber, cocoa, bananas) are situated in the coastal regions of Cameroon. Plantations have several features that suggest they may be hubs of HIV transmission, including: 1) presence of temporary and permanent workers; 2) patterns of in- and out-migration; 3) unbalanced sex ratios (plantation workers tend to be predominantly male as plantation work is perceived to be principally a male activity); 4) workforce characteristics (e.g., generally low educational levels); and 5) rudimentary health and social service infrastructure. In addition, a plantation community is a by-product of an infrastructure created by a corporation with the primary intent of optimizing an agricultural labour force. Given that housing is often provided to plantation workers for extended periods of time in large remote areas, the plantation setting represents a uniquely circumscribed social environment marked by significant cultural, ethnic, and linguistic diversity. The limited available literature suggests plantation work has the potential to transform political, economic, and social roles and responsibilities [8].

Several issues need to be considered when planning research activities in rural plantation settings. From a community engagement perspective, if experience in working with a community is limited, then the potential for mistakes and misunderstandings is likely, including those manifested by unintentional social etiquette blunders and cultural errors [9]. Also, community attitudes and awareness influence participation in research and utilization of programmatic services. Understanding community values is essential to both the development of a research infrastructure and establishment of public trust. Increasingly, public health is seeing a demand for research that is “collaborative and community based rather than merely community placed” [10]. To be of optimal value, this process has to provide an opportunity not only for researchers to understand the community but also for the community to understand researchers and their goal. Beyond familiarizing themselves with a community’s knowledge of and attitudes toward HIV research, researchers have to develop cultural competencies, learn how to foster local capacity building that empowers the community without draining social capital, attain a balance between research goals and community goals, and move from research to action [10,11]. This emphasizes the need for researchers to share their research findings and provide support to participating communities to develop their own HIV interventions and action plans. Finally, investigators must understand the physical conditions, quality of life, social interactions, spatial arrangements, environmental, climatic, and other factors that will influence the research design, behavioural and biological data collection methodologies, as well as data analysis and interpretation.

The intent of our study was to examine facilitators and barriers to planning and conducting HIV research given changing attitudes towards and guidelines for conducting international health research. Facilitators and barriers to HIV research participation are typically examined solely from the perspective of those being researched; however, it is equally important that researchers assess the appropriateness of the physical setting for undertaking complex studies. Thus, an additional goal was to describe the appropriateness of Cameroon agricultural plantations for HIV prevention research, in particular for future cohort studies and clinical trials.

## Methods

Between August and October 2006 we conducted a formative qualitative research study using rapid assessment methods. Qualitative rapid assessment methodologies have proven useful to inform public health interventions involving sexually transmitted infection [11-14].

### Setting

**Figure 1:**
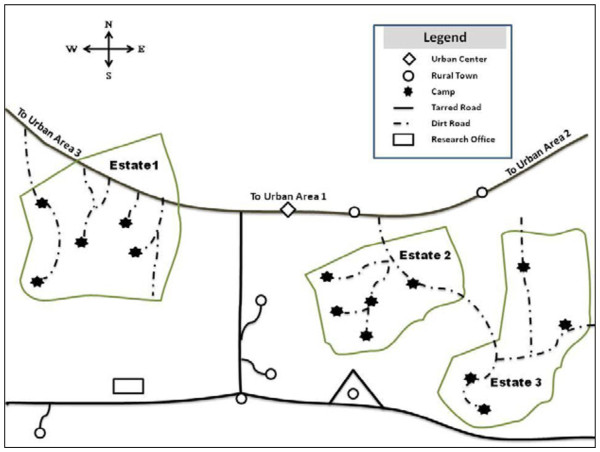
Ethnographic map of 3 plantation estates in Cameroon, August-October 2006.

We conducted the study in three palm oil plantation estates in Cameroon belonging to one of the agricultural companies located in the coastal region. To protect the privacy of the company and the camp communities, the anonym “Company A” is used and the selected plantation estates are referred to as Estate 1, Estate 2, and Estate 3 (see Figure1). "Company A” has five main plantation estates spread across the coastal region of Cameroon (South West and Littoral Regions). Each of these estates is made up of several camps. These camps can include as many as 2250 temporary and permanent workers residing with their families. Temporary workers account for about 80% of the labour force. Women comprise less than 10% of the labour force. Free primary health care is provided to permanent employees (e.g., oil mill operators, agricultural technicians, other technical staff, managers and administrative staff) through company infirmaries located in each estate. Plantation-specific HIV prevalence data are not available. However, free HIV testing, antiretroviral treatment and care are provided to HIV-infected permanent workers at these sites.

Temporary employees and their families are not entitled to receive medical or healthcare benefits. These workers typically seek healthcare from facilities outside the plantation estate. Community research assistants (CRAs) observed that plantation residents typically relied on drugstores, traditional healers, and small medical units situated outside of the plantation camps for their healthcare needs. These medical units were privately operated by unlicensed medical practitioners.

The three estates included in the study were selected because they had available census data, included at least a small female labour force, and had a community leadership (camp administrators and residents’ representatives) that expressed support for HIV research activities. Each estate had 4–5 camps with an average population estimate of 3675 (range 2487 – 4727). Camps were generally 10 to 15 kilometres apart and situated around a central camp that delivered administrative services.

### Procedures

The formative study included structured observations of camp life, as well as group and individual key informant interviews. Here, we apply a classic anthropological definition of a key informant as a community resident who knows the community well and is in a position to broadly discuss its people, practices, values, and services. Group interviews were thus comprised of a collection of key informants.

Seven community research assistants (CRAs) trained in rapid assessment techniques used semi-structured interview guides to facilitate audio recorded group and individual interviews that covered five domains of inquiry: plantation community life; attitudes and behaviours contributing to HIV infection; perceptions about research; barriers and facilitators to HIV research participation; and community involvement in HIV research.

### Structured observations

Because research participants are not always able to describe the communities to which they belong, observation methods become helpful in bridging local narratives with social actions and situations [12,13]. The use of systematic structured observations afforded the opportunity to: a) observe plantation camp residents interact with one another, b) listen to how and when they communicated with one another, c) make sense of the context in which social interactions took place, and identify community features that could either facilitate or hinder HIV prevention research (e.g., medical facility access, distances between camps, road conditions). A minimum of two observations were conducted independently by each of the CRAs over a one-month period in the three plantation estates across 14 camps.

CRAs used a standard observation log to document Spradley’s [15] nine dimensions of descriptive observation: 1) space (layout of the physical setting), 2) actors (information about the people involved), 3) activities (various activities and behaviours of the actors), 4) objects (physical elements such as furniture, tools, etc.), 5) acts (specific individual actions), 6) events (particular events/meetings/ happenings), 7) time (sequence of events), 8) goals (what are actors trying to accomplish), and 9) feelings (emotions displayed in particular contexts). To be as discreet as possible, CRAs took minimal notes during the observation and more detailed notes immediately after an observation was completed.

### Focus group discussions

Because members of the community were familiar with the concept of taking part in group and community meetings, focus group discussions (FGDs) were an appropriate interviewing approach for encouraging them to share their points of view with outsiders. Moreover, this methodology also permitted the research team to generate conversational groups (i.e., small groups that can spontaneously interact in talk that is guided by particular rules and addresses topics in a particular sequence), that made it possible for CRAs to reach observation-like understandings on (1) ways that information was socially introduced and negotiated, and (2) how emphasis on one or more dimensions was used to communicate normative views [16].

### Key informant interviews

We conducted individual key informant interviews (KIIs) for three reasons: 1) not everyone is comfortable speaking in a group setting; 2) a potentially *good* participant (i.e., eloquent and willing to share a rich set of experiences and perspectives) may have special scheduling needs that do not permit him/her to take part in a planned FGD session; and 3) to capture any relevant perspectives or information that were missed by the FGDs, especially information that may not be formally accepted at the normative community level. By collecting data using multiple methods and sources, we were able to compare (triangulate) our major conclusions and thus discern whether they were in agreement [17].

### Participants

A purposive, non-probabilistic sampling approach was used to select all study participants through a combination of nomination and snowball sampling techniques. Gatekeepers (persons, who hold official as well as unofficial positions of authority and access to community resources, that are familiar with the behaviours and lifestyles of the target population) as well as those completing FGDs and KIIs were asked to identify (nominate) persons for study participation. All persons suggested for study participation were screened for eligibility. To be eligible, a person had to: a) have lived or worked at least one year in the plantation community; b) be at least 18 years of age; and c) be a permanent or temporary worker of the plantation or a household member of a plantation worker. Eligible persons received an extended briefing about the study before being asked to provide informed consent. CRAs solicited each key informant’s preference for an FGD or a KI interview and collected contact information that was used solely for scheduling purposes. Separate male and female focus groups were scheduled.

All FGD and KII participants underwent verbal informed consent immediately before initiating data collection. KIIs were conducted in English, French, or Pidgin English. Consent for FGD participants was first administered individually in the language preference of the participant as well as collectively before the onset of the discussion in the language or languages in which participants held fluency. FGDs were conducted in the language of the majority; however, question clarifications and supplemental discussions were carried out in the other two languages when necessary to acknowledge the language and ethnic diversity within the camps. At the end of each data collection session, the interviewer administered a brief demographic questionnaire to each FGD and KII participant. A team consisting of at least one male and one female took part in the data collection, with the person matching the sex of the participant(s) leading the interview. Each FGD and KII took approximately 60 minutes to complete.

The research protocol, verbal consent, and study procedures were approved by institutional review boards at the Centers for Disease Control and Prevention in Atlanta, Georgia and the Cameroon National Ethics Committee. Other than round-trip transport reimbursement in the amount of 1000 CFA (about $2 US), participants received no other monetary compensation for taking part in a KII or FGD. Condoms were provided to all research participants.

### Data analysis

We analysed FGDs and KIIs as a single dataset given that the same type of information was collected regardless of interview type. We applied a two-step analysis process. CRAs held daily debriefing sessions in which they manually categorized information by reviewing the incoming audio recording and interviewer notes into binary matrices (truth tables) to arrive at a snap shot of recurrent themes or concepts for each interview guide item. After all data collection and daily debriefing matrices were completed, matrices associated with each domain of inquiry were grouped together and compared. We identified concepts within the domain that were limited to a single question as well as those that involved multiple questions. A similar comparison approach was used to examine concepts across broader interview guide domains. Context was taken into account to identify how and where conceptual explanations changed. During data collection, these preliminary findings were used to inform the interviewer on additional probe questions for subsequent FGDs and KIIs. Preliminary findings were used to develop the initial codebook for the in-depth analysis.

The second step of the analysis process occurred after all data were collected and involved in-depth computer-assisted textual data analysis. To prepare the data for analysis, CRAs transcribed the English and French audio recordings verbatim. For Pidgin English recordings, a meaning-based translation was prepared. Computer-assisted analysis was done using CDC-developed AnSWR: Analysis Software for Word-based Records Version 6.1 [18]. A standardize coding approach [19] was applied. Transcripts were carefully read through several times by CRAs to identify additional concepts that needed to be defined and included in the codebook. Coding was done in AnSWR by the first author and reviewed by the second author. Thematic analysis was undertaken at two levels, manifest and latent. At the manifest level, ideas that were obvious (i.e., directly observable, explicit) and required little or no interpretation by a coder were coded first. Latent analysis, which followed, required that coders identify underlying ideas and meaning and use information within and across interviews to arrive at an authentic interpretation of the information. Coding discrepancies were discussed and resolved, and the codebook and coding updated where necessary.

After coding was completed, code frequency and code co-occurrence reports were generated to examine coding patterns. For the FGDs the unit of analysis was the group; for the KIIs the unit of analysis was the individual. To avoid over-emphasizing or skewing identification of salient themes (because some participants mentioned a concept more often than others), we looked at code application at unit of analysis level. Multi-dimensional scaling (MDS) and cluster analysis techniques were used to help identify salient themes and patterns. Neither numerical data nor idiosyncratic themes are presented in this paper. Instead, emphasis is on describing recurrent themes and patterns. Quotes, when appropriate, will be used to help illustrate themes and patterns.

## Results

### Sample characteristics

In total, 53 people took part in 6 FGDs (27 men and 26 women), and 12 (6 men and 6 women) in KIIs. FGDs ranged in size from 8–10 participants. The median age for FGD participants was 30 (range = 19 to 56) whereas median age for KIIs was 35 (range = 23 to 48). Sixty-eight per cent of FGD and 83% of KII participants were married (see Table 1). Over 60% of all the study participants were from the Northwest region of Cameroon. The majority had received either primary (40%) or secondary (47%) level education. Only a few (1.5%) had never been to school at all.

**Table 1:** Characteristics of plantation FGD and KII participants in Cameroon, August-October 2006

**Demographic variables**	**Data collection methods**	
	**FGD**	**KII**
	**n = 53 (82%)**	**n = 12 (18%)**
**Site**		
Estate 1	17 (32%)	4 (33%)
Estate 2	18 (34%)	4 (33%)
Estate 3	18 (34%)	4 (33%)
**Sex**		
Men	27 (51%)	6 (50%)
Women	26 (49%)	6 (50%)
**Highest level of education**		
Never attended school	1 (2%)	-
Primary	21 (40%)	5 (17%)
Secondary	26 (49%)	5 (42%)
Post secondary	5 (9%)	2 (3%)
**Marital status**		
Single/never married	15 (28%)	1 (8%)
Married	36 (68%)	10 (83%)
Separated/divorced	1 (2%)	1 (8%)
Widowed	1 (2%)	-
**Work status**		
Permanent salaried worker	1 (2%)	4 (33%)
Permanent fieldworker	11 (21%)	1 (8%)
Temporary worker	9 (17%)	1 (8%)
Self-employed	15 (28%)	-
House wife	6 (11%)	5 (17%)
Student	2 (4%)	1 (8%)
Other	9 (17%)	-
Province of origin*		
Center	6 (11%)	3 (5%)
Littoral	8 (15%)	3 (5%)
Northwest	36 (68%)	3 (5%)
South	1 (2%)	-
Southwest	1 (2%)	-
West	1 (2%)	-
**Interview language preference**		
Pidgin	31(58%)	4(33%)
English	7(13%)	2(17%)
French	15(28%)	6(50%)
**Prior research participation**		
Yes	11(21%)	3(25%)
No	41(77%)	9(75%)
Don’t know	1(2%)	-

*3 KIIs did not provide information on province of origin.

### Community Life

Plantation camps were described as bringing together a multitude of people from different ethnic, religious, tribal, and social backgrounds. Participants reported that English-speaking populations from the Northwest region and French-speaking ethnic groups from the Central, West and Extreme North regions of the country constituted the largest recognizable social groups. Newcomers were reported to be most obvious during holidays when camp members were visited by relatives or at production seasonal peaks when contractors were looking to recruit new hires. Camp residents were depicted as mostly unmarried or as unaccompanied married men who had left spouses behind to seek plantation work. Women without stable partners were referred to as “free”. A workforce comprised largely of men was seen as having an economic draw for women outside of the camps, in particular sex workers. A female key informant explained, “*There are girls who have come in from Douala. They are prostitutes. They come to look for men…. and they go in for them for money. They move from place to place, they do not have a stable place neither do they have any stable man. They are not married. If you offer them any money, they would go in for sexual intercourse or sexual relationship…almost at the end of every month when they are paying workers. They know that when they come they would earn some money and they can even spend a week here and when they have emptied the pockets of their clients they would go back*.”

Participants identified themselves as being relatively unstable and mobile. They described plantation work as tedious and attributed their itinerant lifestyle to dependency on a plantation payment system marked by low and irregular wages. Additionally, participants reported that workers regularly request cash advances against expected earnings from the company, thus pushing them into a cycle of debt. Participants also reported that most plantation work was physically demanding (harvesting, pruning, pesticide application, picking and transportation of palm cones, etc.), therefore making it male-centred and less attractive for women. They stated that women because of their potential for underperformance (i.e., inability to keep up with the physical demands) often encountered hiring discrimination given the company’s concerns about profit margins. Alternating periods of employment and unemployment as well as limited facilities and resources (e.g., housing, schools, and potable water, electricity, and health services) were also said to contribute to the regular movement of people in and out of the plantation community.

CRAs observed that work in the plantations began as early as six o’clock in the morning and ended around two o’clock in the afternoon. Men’s work involved cutting palm cones, pruning fronds, and applying fertilizers and pesticides, while women gathered felled cones and nuts and transferred them to vehicle stations for transportation to processing factories. At the end of their work shift, a number of plantation workers were seen engaging in supplementary income-generating activities. For men, this included raising goats, pigs, or chickens, and tapping palm wine for sale or for personal consumption, trading, distilling and/or selling of locally-made gin known as *ha*. Women generally sold vegetables they brought back from their family farms, and other cooked food items, or sold a locally fermented beer known in the plantations as *sha*. At any time of the day, women could be found decorticating peanuts or pumpkin seeds and processing food either for personal consumption or for marketing around the corridors of their home.

Camp houses were constructed primarily of cement or wood, and had a rectangular uniform design. Wooden houses were occupied by unskilled labourers while the concrete ones housed administrative and technical staff. CRAs also observed across the camps that in many instances, three-to-four families might share a house. In this case, each family occupied a bedroom and used the living room as communal space. A female key informant states, “*When new faces [people] come they do not have houses to stay because the houses are not enough and if they have a relation they would crowd there and the relation would not be able to sleep well. And at times when the house is so crowded feeding becomes a problem. It could have about 7 – 8 people. I have said some houses have one room, others about two rooms and a parlour, and the upper camps have about three rooms and a parlour. The houses given by contractors are a room to each person. They give them houses but they are many in a room.*”

Leisure activities, for the most part, were seen to occur in late afternoons, evenings and weekends (i.e., non-work time). Leisure activities included both indoor and outdoor games, TV viewing, radio listening, talking, attending or participating in soccer and other field sports, and going to video clubs (commercial spaces purposely designed for viewing, at a fee, video films). Within each of the camps at least one bar existed, but in most several bars and open places designated for drinking and social interactions were present. The combination of large numbers of people, bars and market outlets, as well as the availability of electricity, sometimes pipe-borne water, and playgrounds, appeared to draw adjacent non-plantation populations into the camps during leisure hours. Dancing spots were available in some of the camps, but in most cases bars had room for dancing. The entertainment allure to non-plantation populations is described by a male key informant in the following way: “*Some people come here to visit their relatives. Others come here during pay days. Many visitors come here to enjoy with us on pay days….Many of them are attracted by money…. . We are very simple, we like to entertain visitors, give them food and drinks, we do not send away visitors….They come here because the camp is the same like the village, we have off licenses, and we have clubs. Most of the clubs are opened on pay days and on quinzieme (day of advance payment).*

### Knowledge and concerns about HIV

While participants indicated that their communities were being negatively affected by HIV and other sexually transmitted diseases, they were more likely to voice health concerns about malaria, gastrointestinal conditions (e.g., diarrhoea, typhoid, worms, gastritis, dysentery), yellow fever, and skin diseases and disorders (e.g., filaria, fungi, scabies, eczema). Participants indicated that low awareness or knowledge about HIV as well as of sharp objects (blades, needles, shaving machines, syringes) increased risk for HIV transmission and acquisition. Unprotected sex with multiple partners, however, was viewed as the primary reason for people in the camps becoming HIV infected. Participants explained that the disproportionate number of men in the camps was the cause of frequently overlapping sexual relationships where the sharing of female sexual partners was mutually and socially tolerated or benignly overlooked. In some instances, sharing of partners was in the context of prostitution, which was also identified as a key contributor to the spread of HIV. A male focus group participant stated, “*In this community, we have many women here who depend on sex to pay their house rent, food and other things. We simply mean that, there are women who, after six or seven o’clock, they take their bath and move around to look for boys who can propose them 1000, 500 or any amount of money….Most of them are prostitutes. They only depend on boys’ money to pay their rent and other things….Here we have about 700 boys for 200 girls*”.

The ABC prevention messages (abstinence, be faithful, use condoms) resonated in participants’ responses about what plantation workers and residents could do to avoid HIV infection. They, however, acknowledged that it was difficult to adhere to such principles and practices. They mentioned that condoms were not readily available in the camps. Moreover, condoms were viewed as decreasing sexual pleasure, being tainted with HIV (in particular those containing lubricant), and not truly reliable in preventing sexually transmitted diseases. Study participants further described condom use as complex, explaining that an overriding sexual urge at foreplay often overshadows the necessity for condom use and risk reduction during sexual encounters. One male focus group participant explained, “*I will talk as one of the young people who like to participate in those kinds of things. As we do here, if we see a new girl, we try to contact her and bring her closer to us and when our mission is fruitful we have sex with her. So when we are going to have sex with her, we don’t even know how far she came, we don’t know which kind of disease she brought, and we also do not have the time for condom. Because we have shortage of girls, you will only hurry to sex and go, and sometimes since it is difficult to get girls, another boy may see her coming out of your house or room and he too will take her and go with her to his own corner……. The girl can have sex with 10 men as I earlier said. So we don’t know the kind of disease that she can bring and distribute to us.*”

Another male focus group participant indicated that “*abstinence which has to do with love is one of the most difficult things to observe; this is because love always ends with sex. And for the use of condom….in French they say ça freine le mouvement -- it obstructs movement, which is like it reduces pleasure. So I think that is the hardest…. The sexual aspect is difficult because they feel when they use a condom they do not experience the real thing.*”

### Perceptions of research and HIV research participation

Before sharing their thoughts on facilitators and barriers to taking part in HIV research, participants were asked to provide their definitions of the word research. Understandings of the term ranged from an abstract concept (“*know more about something*”) to a more applied one (“*looking for ways to prevent health problems” or “bringing development or improvement*”). Overall, one-fourth of study participants reported prior participation in research. The remaining participants indicated that familiarization with public health interventions, namely HIV sensitization and immunization campaigns, was more common. For some, the distinction between participation in public health interventions and research was unclear. Within this context, general attitudes towards HIV research centred around three key themes: hope, opportunity, and wariness (Table 2). While HIV research was viewed as promising in treating (curing) and ultimately eliminating the spread of the virus and potentially improving normative knowledge and attitudes about the disease, participants voiced scepticism about the practical value of research outcomes, specifically in terms of the availability, access, and sustainability of treatment services.

**Table 2:** FGD and KII participant’s perceptions about community-held attitudes toward HIV research express1ed by plantation FGD and KII participants, August-October 2006

	**Hope**
·	Eliminates or reduces people’s fears of dying from AIDS
·	Discovers a cure for HIV or a means of preventing one from becoming infected
·	Improves drugs to treat HIV/AIDS
	**Opportunity**
·	Improves community understanding of HIV
·	Removes social threat of HIV diagnosis
·	Provides care for HIV infected persons
·	Enables the exchange of ideas between researchers and the community
	**Wariness**
·	Has no sustainability (leaves no representative behind to keep people informed)
·	Fails to reach people because they are busy working (have no time or are too tired to participate)
·	Has no practical value to the community (does not believe that HIV or AIDS exists)
·	Uses people solely for experimental purposes (i.e., ‘*guinea pigs’*)
·	Has no means of making HIV testing and antiretroviral drugs available to the community at an affordable cost

### Community participation facilitators

Participants also stated that HIV research should focus on improving the general welfare of the community as opposed to restricting itself to merely extracting information from it. As stated by a female key informant, “*Research participants want to feel they are better off than they were before research was introduced to them*.”. Another male focus group participant reflected that community rejection of HIV research may result from a perception that this is just a “*dry academic exercise*”.

Protecting the personal information of participants, especially those infected with HIV, was also regarded as a strategy that could encourage participation in HIV research in their community particularly if HIV testing is part of the research procedures. Study participants recommended that other diseases such as malaria, typhoid, and STIs be integrated into the HIV research process to reduce stigma for participants.

Participants indicated that researchers should expect to provide incentives at both the individual- and community-level. The absence of incentives or anything of immediate benefit to participants was perceived to discourage participation. Recommended individual incentives included provision of free drug samples, condoms, and monetary reimbursement. Participants framed suggestions for community-level incentives based on their personal assessment that the conditions at camp health facilities were unacceptable, thus improving the health infrastructure with quality services would likely motivate many to take part in research.

Participants indicated that HIV research that took into account community insights as part of the planning and implementation process could better integrate critical elements of respect, trust, empathy from researchers, and meaningful communication. Participants remarked that broad-based acceptability of research in their community will depend on how researchers communicate their goals to the community. Finally, acceptance of HIV research in plantation communities was viewed as requiring assurances that disruptions to “normal everyday life” would not occur.

### Community participation barriers

Participants’ responses regarding barriers to HIV research participation centred on HIV testing issues. We identified five subthemes associated within a broader theme of HIV testing fears: (1) apprehension that test results were likely to show infection; (2) dismay that people would be unable to cope with receiving HIV-positive or conflicting test results (i.e., unmatched results from parallel rapid HIV test); (3) concerns that HIV testing would be forced upon research participants against their will; (4) unease that those taking part in the research would be required to personally cover the cost of expensive HIV tests; and (5) worries that HIV testing efforts were being disguised as research.

The time-related costs were also considered as possible deterrents to research participation. It was perceived that people would not take part in research if it interfered with their work schedules, was considered a waste of time, or took them away from other responsibilities (e.g., their farms). In one female focus group, we heard that “*research is for idle people*” and without reimbursement for transport and other incentives “*people preferred to stay idle in their houses rather than go there and waste their time*”.

Participants indicated that prior negative interactions with healthcare providers and staff might discourage some plantation members from taking part in HIV research. They often talked of their own or other’s experiences where healthcare workers had shared personal and confidential information with others in the community. Several indicated that “*it is necessary to be discreet*” and to know how to keep participants information “*secret*”. Some participants said that “*after having done the test, one [the doctor] starts to speak badly about you*”. Moreover, concerns that care and treatment services would not be available, were costly, or were of poor quality were also discussed.

Lastly, we identified two barriers that focused on behaviour. Participants expressed concern that researchers would encourage, if not outright require, that persons enrolled in studies take part in HIV-risk behaviours (e.g., adopt or maintain a sexually promiscuous lifestyle) to help determine whether an intervention effectively prevented, treated, or cured HIV infection. Concerns were voiced that people would be experimented on and treated as research guinea pigs. One male FGD participants stated,

*« For example, I do the AIDS test, and I am told that I am seropositive, then I am left like that and I go home !…..I will prefer that after I test positive, you should call me and say, ‘my brother as you are positive like this, this is not the end of the world. Life is still going to continue for you, you are going to live. And then you do something to safe my life.’ So this is where the problem is, people want to feel secured”*.

Conversely, some participants stated that requiring people from abstaining from alcohol use and sex would likely discourage research participation. Table 3 summarizes key facilitators and barriers to research participation identified by study participants.

**Table 3:** FGD and KII participant’s perceptions on what their communities would view as facilitators and barriers to HIV research participation, August-October 2006

	**Facilitators**
·	Improves general community welfare
·	Provides comprehensive health services by offering care and treatment for common illnesses
·	Protects personal information
·	Provides incentives
·	Incorporates community input in the planning and implementation phases
·	Avoids or minimizes disruptions to ‘everyday life’
	**Barriers**
·	Further fuels fears of HIV test
·	Mistrust of researchers (treated as guinea pigs; fail to keep participant information confidential and private)
·	Requires people to take time away from work or other important activities
·	Access to medical care and treatment difficult or costly, or care and treatment is second-rate
·	Promotes risk-taking behaviours
·	Requires abstaining from alcohol use and sex

## Discussion

In this study, we used a culturally appropriate engagement approach that is grounded in community perceptions, beliefs and expectations. We found this approach encouraged participants to share information about themselves and their community. Interacting with the community, learning and understanding their perceptions and preferences provided valuable means of engaging the community and maximizing the potential for community participation. The importance of community participation in HIV research has been demonstrated across different settings and is considered “good participatory practice” by UNAIDS [20]. Working in Uganda, Kiwanuka-Tondo and Synder [21] found that organizational characteristics and quality of community engagement had a great impact on peoples’ willingness to participate. Manson and colleagues [22] found that cultural values necessary for performing research with indigenous populations revolved around “trust, respect, self-determination, mutuality of interest, perspective taking, full participation, reciprocity, collective benefit and long-term commitment” (p. 73 S). Averill [23] concluded that without effectively engaging the community through building trust and reciprocity, researchers may end up with irrelevant findings and recommendations. UNAIDS guidelines for good participatory practice also invokes core principles of respect, autonomy, transparency, and access to care [20].

That study participants place a high premium on politeness, trust, respect, communication, tolerance and empathy from researchers towards their community suggests that some of the fundamental barriers to HIV research participation may lie in researchers’ untoward attitudes, behaviours and manner of interaction with their host communities. Notably, participants indicated that researchers should respect confidentiality and protect the privacy of HIV-infected participants. These findings are consistent with revelations from other studies and reinforce the UNAIDS core principles.

Our research was conducted against the backdrop of the premature closure of a Phase III clinical trial of antiretroviral pre-exposure prophylaxis among female sex workers in Douala [24]. This closure was driven by community and international advocate group concerns about incomplete risk communication as well as incomplete informed consent and post-trial care for women who tested HIV positive. These controversies drew extensive domestic and international media coverage. During the same time period, there was heightened attention in the international research community about the conduct of scientifically and ethically sound multinational HIV research, particularly in developing countries, and UNAIDS was in the process of developing new guidelines for “good participatory practice” for biomedical HIV prevention trials [20].

Interestingly, our study participants made no mention of this study despite the plantation’s geographic proximity to the city where the trial was being conducted. This could be interpreted as evidence of the relative isolation of these plantation communities.

Elsewhere in Kenya and Uganda, studies found that study participants most commonly indicated that they desired a “kind” person to conduct voluntary counselling and testing [25]. Further, in Thailand and also in Kenya, study participants showed an overwhelming willingness to participate in future HIV prevention research but this initial willingness could be compromised if the research process failed to adequately deal with community-specific expectations, sensitivities and other social complexities [26,27]. Our study not only lends support to these views but also underscores the need for researchers to understand communities and let communities understand researchers as a critical means of establishing trust, promoting informed participation in HIV research at the individual- as well as community-level, and share in the understanding that addressing HIV prevention and treatment is a shared endeavour. This has the potential to minimize research-related rumour, controversy and failure.

Participants cited several themes that would suggest increased risk of HIV transmission in this community, including: concurrent sexual relationships; sex in exchange for money or other resources; scarcity of condoms; reluctance to use condoms because of perceived reduction in pleasure and lack of confidence in their protective effects; difficulty abstaining from sex; and commonly encountered sexually transmitted infections (STIs). From the reports of study participants, members of the plantation community desired access to general health services such as prevention and treatment of malaria and gastrointestinal disorders, voluntary HIV counselling and testing, provision of condoms, and comprehensive HIV/STI prevention, care and treatment programs. Access to general healthcare services was viewed as a critical community benefit, and a precondition for long-term research to take place in this low-resource setting.

We found that providing modest incentives (transport reimbursement, condoms) was valuable to the process of engagement and provided tangible thanks for the contributions participants made to the study and data generation process. We also found that these minimal incentives kindled community’s enthusiasm for our project and increased desire to participate in HIV research. Incentives have proven to be an effective tool in reducing refusal as well as discontinuation rates among research participants in developed and developing countries [28]. Since participants often incur costs for their participation, providing incentives could help to defray some of these costs. Our study provides additional evidence that incentives are critical for community engagement and maximizing research participation even in settings that are relatively research-naïve.

This study reveals the presence of multiple barriers, real or perceived, that could potentially affect participation in HIV research in rural agro-plantation communities in Cameroon. Alternating periods of employment and unemployment and regular migration of people into and out of the plantation community make this population an unsuitable target for longitudinal public health HIV investigations. Longitudinal studies targeting this community would have to establish a means to track temporary workers outside of the plantation setting, or may have to exclude these workers, who constitute about 80% of company employees, due to their propensity to be mobile.

One important factor likely to limit participation in HIV research among these workers is the pragmatic issue of time, inconvenience and the probable disruption to normal daily routines resulting from their participation. Plantation workers in this setting have full schedules since wages depend on the amount of labour accomplished and number of hours worked. To maximize their earnings, workers often have to work overtime, and thus have little time for other duties. Providing incentives can also be a symbolic compensation for work hours lost due to participation and could minimize the direct and indirect cost of participation.

Although most study participants had not previously participated in research, some of the potential barriers they identified were similar to those cited by persons in more research-familiar settings. Qualitative studies have found that fear of being treated like guinea pig, [29,30] uncertainty about research outcome for participants [29] inadvertent disclosure of participants’ HIV status [31], and pragmatic obstacles such as changes to daily routines [32] could be major barriers that minimize people’s participation in research. Our findings complement observations from Kenya, South Africa and Thailand where systematic reviews and other studies cited mistrust of researchers and the research process as significant barriers to participation in HIV research [26,27,33] .

Our study is not without limitations. Although conditions in our study setting have changed little, if at all, a 6-year time lapse from when the data was collected to when it is published is in itself a limitation worthy to be noted. As in most qualitative studies, our sample size was small. Our findings may be unique to those persons taking part in this formative study and therefore not generalizable to the larger plantation population or other settings. Moreover, perceived facilitators and barriers to HIV research participation may differ greatly from those actually experienced. Nevertheless, the study offers insights on potential facilitators and barriers to participation in HIV research among these plantation communities. As a reflection on “good participatory practices”, the study has also been able to demonstrate the importance of formative work prior to initiating HIV prevention research in a unique community setting.

## Conclusions

Our study shows that consistent with internationally accepted good participatory practices for HIV prevention research, formative research can be used both as a tool for engaging the community and for identifying potentially needed services that are desired by the community. Community members viewed access to improved general healthcare including HIV services as an essential community-wide incentive prior to the initiation of longitudinal research in this underserved and remote setting. While the intent of this study was not to assess whether high HIV risk behaviour was present in plantation communities, our data suggest that HIV prevention and health promotion program interventions such as voluntary counselling and testing for HIV, condom provision, and access to comprehensive medical care including HIV and STI treatment are sorely needed in these communities.

## Abbreviations

ABC, Abstinence, be faithful or use condoms; AnSWR, Analysis Software for Word-based Records; CDC, Centers for Disease Control and Prevention; CRAs, Community research assistants; FGD, Focus group discussion; HIV, Human Immunodeficiency Virus; KII, Key informant interview; STIs, Sexually transmitted infections; UNAIDS, Joint United Nations AIDS Program.

## Competing interests

The authors declare that they have no competing interests.

## Authors’ contributions

EK and EML contributed to the formulation of the study and co-wrote drafts of the manuscript. JM, KC, and PR also contributed to the formulation of the study, reviewed, and provided substantial inputs into the manuscript. All the Authors read, provided substantial input and approved the final manuscript. EK and EML are guarantors of this paper.

## Pre-publication history

The pre-publication history for this paper can be accessed here:

http://www.biomedcentral.com/1472-698X/12/8/prepub
